# scZAG: Integrating ZINB-Based Autoencoder with Adaptive Data Augmentation Graph Contrastive Learning for scRNA-seq Clustering

**DOI:** 10.3390/ijms25115976

**Published:** 2024-05-29

**Authors:** Tianjiao Zhang, Jixiang Ren, Liangyu Li, Zhenao Wu, Ziheng Zhang, Guanghui Dong, Guohua Wang

**Affiliations:** College of Computer and Control Engineering, Northeast Forestry University, Harbin 150040, China; tianjiaozhang@nefu.edu.cn (T.Z.); 1241126773@nefu.edu.cn (J.R.); liangyuli@nefu.edu.cn (L.L.); 2022112562@nefu.edu.cn (Z.W.); zzhjs@nefu.edu.cn (Z.Z.); dongguanghui@nefu.edu.cn (G.D.)

**Keywords:** scRNA-seq data, APPNPGCN, graph contrastive learning, ZINB model, KL divergence

## Abstract

Single-cell RNA sequencing (scRNA-seq) is widely used to interpret cellular states, detect cell subpopulations, and study disease mechanisms. In scRNA-seq data analysis, cell clustering is a key step that can identify cell types. However, scRNA-seq data are characterized by high dimensionality and significant sparsity, presenting considerable challenges for clustering. In the high-dimensional gene expression space, cells may form complex topological structures. Many conventional scRNA-seq data analysis methods focus on identifying cell subgroups rather than exploring these potential high-dimensional structures in detail. Although some methods have begun to consider the topological structures within the data, many still overlook the continuity and complex topology present in single-cell data. We propose a deep learning framework that begins by employing a zero-inflated negative binomial (ZINB) model to denoise the highly sparse and over-dispersed scRNA-seq data. Next, scZAG uses an adaptive graph contrastive representation learning approach that combines approximate personalized propagation of neural predictions graph convolution (APPNPGCN) with graph contrastive learning methods. By using APPNPGCN as the encoder for graph contrastive learning, we ensure that each cell’s representation reflects not only its own features but also its position in the graph and its relationships with other cells. Graph contrastive learning exploits the relationships between nodes to capture the similarity among cells, better representing the data’s underlying continuity and complex topology. Finally, the learned low-dimensional latent representations are clustered using Kullback–Leibler divergence. We validated the superior clustering performance of scZAG on 10 common scRNA-seq datasets in comparison to existing state-of-the-art clustering methods.

## 1. Introduction

Single-cell RNA sequencing (scRNA-seq) is a formidable tool that provides in-depth insights into the genetic traits of each individual cell [1]. This technology is invaluable for distinguishing cell types with fine granularity [2], exploring the depths of developmental biology, identifying the mechanisms of complex diseases [3], and mapping the developmental trajectories of cells [4]. In the analysis of scRNA-seq data, precisely discerning different cell types is an essential step [4]. As such, cell clustering methods have become a crucial component of scRNA-seq data analysis, capable of identifying a variety of cell types without any preset assumptions [2]. Traditional clustering techniques, such as K-means [5], hierarchical clustering [6], and density-based clustering [7], have been utilized to tackle clustering tasks. Nonetheless, the clustering analysis of scRNA-seq data still presents computational and statistical challenges, as limitations in sequencing technology and environmental factors lead to extreme sparsity in the data and a high incidence of zeros [8,9]. Therefore, the development of more efficient and accurate clustering methods for scRNA-seq data is compellingly necessary.

Numerous clustering methods have been developed to address these challenges. For instance, CIDR employs a fast PCA-based approach using dissimilarity matrices for data imputation and clustering [10]. SC3 introduces a consensus clustering framework tailored for scRNA-seq data, reducing dimensions through PCA and Laplace transforms [11]. SIMLR utilizes multi-kernel learning to attain more robust distance metrics and to handle extensive data missingness [12]. Despite these advances, due to the sparsity of gene expression levels in the data, these methods often provide suboptimal solutions when dealing with scRNA-seq data [13]. Furthermore, these methods commonly rely on computationally intensive full-graph Laplacian matrices, which demand considerable computational and storage resources. AutoClass learns the data distribution from raw scRNA-seq data and reconstructs gene expression values based on specific cell types. However, these methods rely on the original distribution of scRNA-seq data and overlook the topological structure information inherent in the data. In recent years, deep embedding clustering methods have emerged as successful approaches for modeling high-dimensional and sparse scRNA-seq data. Examples include scDeepcluster and scziDesk. These methods refine clusters iteratively by learning highly confident assignments and leveraging auxiliary target distributions, ultimately leading to improved clustering results. Nevertheless, these deep embedding clustering methods frequently overlook the propagation of structural information and the relationships between nodes.

Recently, graph neural networks (GNNs) have garnered attention from researchers due to their ability to capture the relational information between neighboring nodes in a graph [14]. GNNs can reveal the connections between a target node and its surrounding nodes, thereby enhancing the representation of node features [15]. This has made GNNs a popular method for processing single-cell RNA sequencing (scRNA-seq) data. For example, scGAE utilizes a graph autoencoder to preserve the topological structure and perform dimensionality reduction on scRNA-seq data. GraphSCC combines graph convolutional networks with denoising autoencoder networks to simultaneously capture the complex relationships between cells and the intrinsic characteristics of cells. scTAG leverages GNNs to summarize the related data of adjacent nodes and maps cell expression data to a ZINB model [13]. Although current GNN-based methods have achieved remarkable results in clustering scRNA-seq data, these strategies often overlook the global information of the graph, leading to an inability to better extract effective latent features.

Due to the limited and hard-to-obtain labeling resources for cell types, as well as the difficulty in learning more effective feature representations, graph contrastive learning has demonstrated strong potential. The core idea is to improve the accuracy of feature representation by increasing the similarity between positive samples while decreasing the similarity between negative samples. Contrastive learning is mainly used in unsupervised representation learning, where it can fully utilize a large amount of unlabeled data. It has shown superior performance on these datasets, even surpassing some supervised learning methods [16]. This makes contrastive learning naturally suited for scRNA-seq data analysis. The first method to apply this to scRNA-seq data clustering was contrastive-sc [17], which uses a dropout neural network layer to randomly mask a set of genes, assigning a weight of zero to randomly selected genes. In this case, some key features may be ignored, and important features for model learning might be missed, potentially leading to decreased accuracy in clustering [16]. scNAME [18] combines neighborhood contrastive loss with an auxiliary masking estimation task to delve deeper into the correlations between features and similarities between cells. While common contrastive learning methods effectively utilize genes as features of cells, they do not consider the interrelations between cells. GNNs, however, have the capability to capture and represent the complex high-order structural relationships between cells [19]. Therefore, using graph contrastive learning for cell clustering is a novel approach that could potentially improve the accuracy of cell clustering.

Consequently, we introduce a novel deep graph contrastive learning clustering method called scZAG. scZAG utilizes a ZINB graph convolutional autoencoder to capture the zero-inflation characteristic of scRNA-seq data and reduce noise impact. It employs a joint adaptive data augmentation strategy targeting topological structures and node attributes, preserving key structures and attributes within the cell graph. Utilizing an APPNPGCN as the encoder, scZAG effectively captures local and global graph structure information. By leveraging graph contrastive learning, scZAG learns representative node features and optimizes the cell clustering process using Kullback–Leibler (KL) divergence, ensuring similar cells are assigned to the same cluster while dissimilar ones are separated.

## 2. Results

### 2.1. Implementation Details

In our scZAG method, the construction of the cell graph utilizes the KNN algorithm, with the hyperparameter K set to 15. For the APPNPGCN autoencoder segment, we designed two layers with 128 and 15 nodes, respectively. Concurrently, the fully connected decoder comprises three hidden layers, with node counts of 128, 256, and 512 in sequence [13]. We employ the Adam optimizer to train the model, which is divided into two parts: pre-training and main training [20]. In the pre-training phase, we set the model to run for 1000 epochs with a learning rate of 1 × 10^−4^. In the subsequent main training phase, the model undergoes 300 epochs, with the learning rate adjusted to 5 × 10^−4^. For the baseline methods, we adhere to the hyperparameter settings, as reported in the original publications. All our experiments were conducted on a CentOS 7.9 server equipped with an Intel(R) Xeon(R) Gold 6326 CPU @ 2.90 GHz, two NVIDIA A100 GPUs each with 40 GB of memory, and 1 TB of RAM.

### 2.2. Clustering Performance

We compared five state-of-the-art scRNA-seq clustering methods, including scziDesk [21], GraphSCC [14], scGAE [22], scDeepCluster [23], and AutoClass [24].

**Figure 1:**
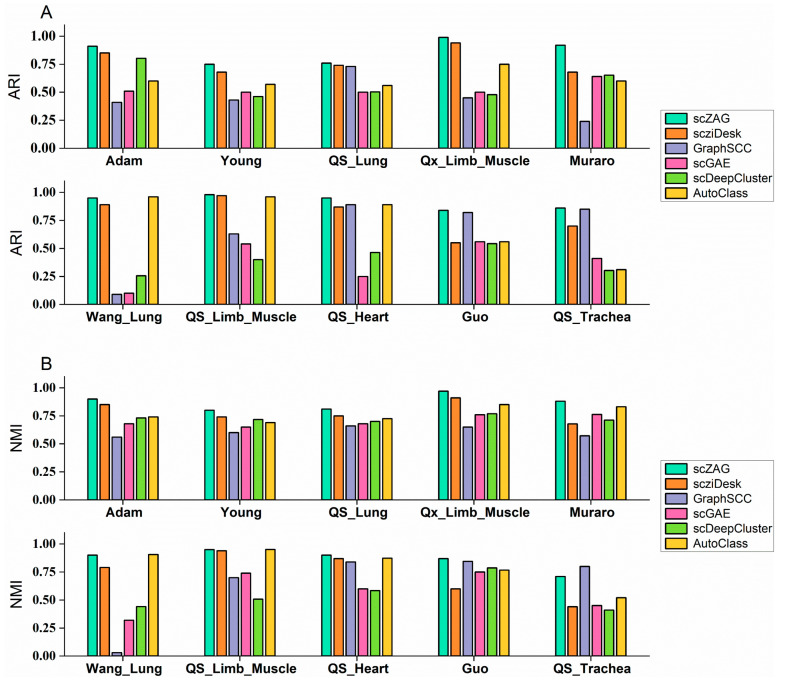
ARI (**A**) and NMI (**B**) scores of scZAG and five other clustering methods across ten real-world datasets.

As illustrated in Figure 1, our method, scZAG, was compared across 10 datasets, with all experimental results obtained by averaging five runs for each method. In the comparative experiments across these datasets, scZAG achieved the highest ARI scores on nine datasets and the highest NMI scores on eight, thus demonstrating the superior performance of our method among those evaluated. The figure reveals that the clustering performance of the other methods was not consistent, with inherent limitations restricting their generalizability. From Figure 1, it is evident that scDeepCluster performs poorly on most datasets due to its neglect of the underlying relationships between cells. Utilizing graph neural networks enables the capture of relational information between adjacent nodes in the graph, thereby enhancing the representation of node features. However, we observed that conventional deep graph embedding models did not exhibit advantages, and their clustering performance was not sufficiently stable. For instance, in the “Wang_Lung” dataset, the presence of only two cell types may lead to a biased and highly sparse data distribution. Consequently, this could result in suboptimal performance for methods like GraphSCC and scGAE, which rely solely on the information structure retained in the cell graph and may struggle to accommodate the peculiarities of scRNA-seq data. Consequently, further simulation of the data through the ZINB distribution was necessary, once again highlighting the superiority of scZAG. Additionally, employing deep embedded clustering methods with the ZINB distribution model, including scziDesk and scDCC, demonstrated better and more stable clustering performance. Although scziDesk and AutoClass achieved commendable performance on certain specific datasets, they both overlooked the transmission of information between cells and the relationships among them. Our use of APPNPGCN allows for the integration of local information from neighbors and global information from more distant nodes, enabling a better grasp of intercellular information transmission; the use of graph contrastive learning captures the complex relationships between cells more effectively, leading to more accurate cell type distinctions.

**Figure 2:**
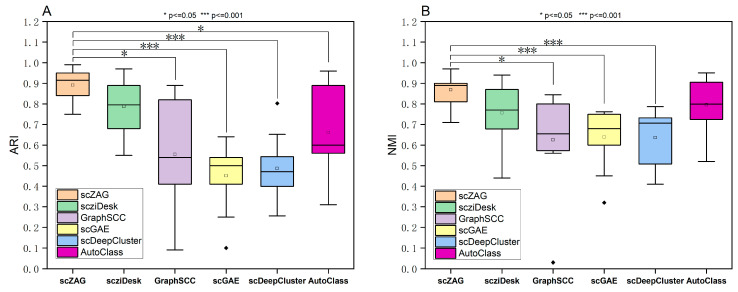
Box plots for six methods across ten datasets, along with significance analysis, are presented. This analysis is presented for the ARI (**A**) and NMI (**B**) metrics.

To further substantiate the superiority of scZAG over other methods, we conducted a Mann–Whitney U test, also known as the Wilcoxon rank-sum test, as shown in Figure 2. The Mann–Whitney U test is a non-parametric statistical method that provides a means to compare the medians of two independent samples without relying on specific distributional assumptions. The statistical outcome produced by the test, namely the *p*-value, allows us to definitively determine the presence of significant differences between scZAG and the other four methods. From Figure 2, it can be observed that when the *p*-value is set to 0.05, significant differences exist between scZAG and both GraphSCC and AutoClass in terms of the ARI evaluation metric. Similarly, significant differences are observed between scZAG and AutoClass in terms of the NMI evaluation metric. However, when the *p*-value is set to 0.001, significant differences are found between scZAG and both scGAE and scDeepCluster in terms of both the ARI and NMI evaluation metrics. These results indicate significant discrepancies in clustering performance between scZAG and the other algorithms at different significance levels, demonstrating superior clustering performance of scZAG over other methods. Moreover, it can be further elucidated that scZAG effectively performs dimensionality reduction of scRNA-seq data by utilizing the ZINB-based autoencoder. By leveraging a joint adaptive data augmentation strategy targeting topological structures and node attributes, scZAG preserves key structures and attributes within the cell graph. Through graph contrastive learning on the two enhanced cell graphs obtained from the augmentation strategy, scZAG learns representative node features, thus improving clustering accuracy. By effectively addressing the challenges of high dimensionality and high dropout events in scRNA-seq data, scZAG consistently demonstrates outstanding performance across multiple datasets and evaluation metrics, thereby assisting researchers in tasks such as cell type identification in single-cell RNA sequencing data.

### 2.3. Visualized Analysis

**Figure 3:**
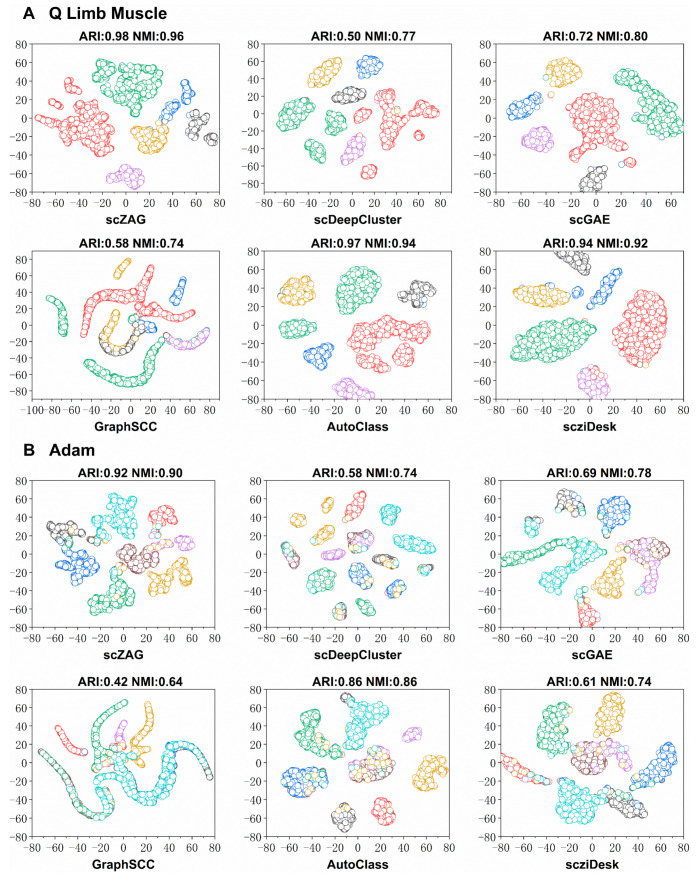
Visualization analysis of cell types. (**A**) Clustering of the Qx_Limb_Muscle dataset using six clustering methods, followed by visualization with 2D t-SNE. (**B**) Clustering of the Adam dataset using six clustering methods, followed by visualization with 2D t-SNE.

To demonstrate the intuitive clustering performance of scZAG, we selected two real datasets (Qx_Limb_Muscle and Adam) with different sample sizes and subtypes for cell visualization. From Figure 3, we can observe that scZAG displays distinct cell clusters with clear boundaries compared to other methods. scZAG distinctly separates more cell clusters, and the clustering results are more accurate, with each cluster containing fewer cells of other types. The scDeepCluster and scGAE methods incorrectly split cells of the same type into multiple clusters. GraphSCC fails to clearly distinguish between cell clusters. On the other hand, the clusters identified by AutoClass and scziDesk contain a large mixture of other cell types, leading to lower accuracy in the results. These visualizations provide a vivid corroboration of our numerical findings, clearly demonstrating that scZAG can not only accurately identify various cell types but also tightly group cells of the same type together while effectively distinguishing between different cell types. Through visual analysis, we can effectively demonstrate that scZAG outperforms other methods in terms of visualization quality and clustering performance. Compared to other clustering methods, the clusters generated by scZAG are clearer, more compact, and exhibit more distinct separation between clusters.

### 2.4. Parameter Analysis

**Figure 4:**
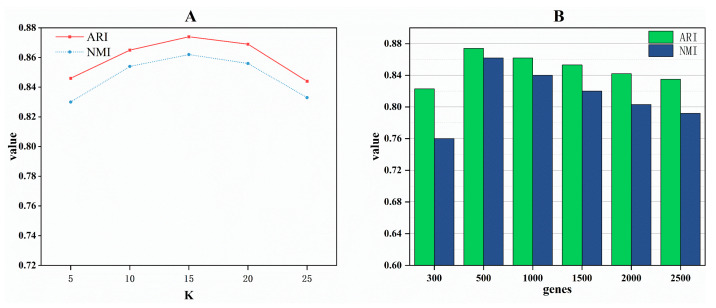
Parameter analysis. (**A**) The impact of different neighbor parameters K on ARI and NMI values. (**B**) The effect of varying numbers of highly variable genes on ARI and NMI values.

**Influence of the Hyperparameter K in the KNN Algorithm:** In constructing cell neighborhoods using the KNN algorithm, K represents the number of nearest neighbors for each cell. To select an optimal value for K, we experimented with different K values to observe their impact on downstream analysis results. As illustrated in Figure 4A, both evaluation metrics peak when the hyperparameter K is set to 15. Consequently, we designated 15 as the hyperparameter K within our scZAG model.

**Analysis of the Number of Highly Variable Genes:** In scRNA-seq data analysis, highly variable genes are crucial for distinguishing cell types or states due to their significant expression variations across different cells. Focusing on these genes reduces data dimensionality and emphasizes the most informative ones. In our model, we determined the optimal number of highly variable genes through multiple experiments, ranging from 300 to 2500. Figure 4B illustrates the average NMI and ARI for selecting 300, 500, 1000, 1500, 2000, and 2500 highly variable genes across 10 datasets. Optimal values for both metrics are observed when selecting the top 500 highly variable genes. Therefore, in our scZAG method, we proceed with experiments using the top 500 highly variable genes.

### 2.5. Ablation Study

To assess the contribution of each component to our scZAG model, we carried out ablation experiments under three scenarios: (1) excluding APPNPGCN, where we substituted the APPNPGCN with a conventional GCN; (2) excluding the adaptive graph contrastive learning (GCA) module while keeping the rest of the components intact; and (3) excluding the ZINB model, again with the rest of the components remaining unchanged. Table 1 presents the mean ARI and NMI scores achieved by scZAG on ten distinct datasets. As per the table, it is evident that each component positively influences the overall performance of scZAG. The APPNPGCN, utilizing power series and random walks for feature propagation, effectively spreads information across the graph, which is conducive to feature integration between cells and enhanced clustering. The graph contrastive learning module is designed to discern the similarities and differences among nodes, helping to identify and accentuate those pivotal features that are instrumental in differentiating cell types. Lastly, the ZINB model captures the zero-inflation aspect of scRNA-seq data, which tailors our method to the data more aptly, thereby enabling more accurate delineation of cell subpopulations in clustering outcomes.

## 3. Discussion

We have introduced a deep learning method for scRNA-seq clustering named scZAG. Although traditional graph neural networks excel at processing graph-structured information, they can sometimes be overly influenced by the graph structure, especially when the edge weights are not set properly. To overcome this issue, scZAG incorporates APPNPGCN, a method that achieves a better balance between node features and graph structure information through an improved propagation scheme based on personalized PageRank. Given that scRNA-seq data typically contain multiple cell types and states, which present complex patterns and structures in high-dimensional space, the iterative propagation mechanism of APPNPGCN aids the model in capturing the intricate relationships between these cells.

Furthermore, we have implemented a novel adaptive data augmentation approach. By integrating graph contrastive learning, this method is designed to learn the similarities and differences between nodes, aiding in the identification and emphasis of key features that facilitate the distinction of cell types or states, thereby enhancing the clustering outcome. To further augment the model’s performance, we have integrated a ZINB model within the APPNPGCN encoder. The ZINB model handles the zero-inflation and discrete distribution commonly found in scRNA-seq data, providing a more accurate representation of gene expression profiles. By integrating the ZINB model with GNN, we can leverage the more precise cell feature representations provided by ZINB. This enables GNNs to learn more discriminative feature representations, thus improving the clustering algorithm’s ability to identify cell types and states accurately. Personalized PageRank, combined with the ZINB model, offers more accurate information propagation by incorporating both graph structure and cell features. This integration allows clustering algorithms to utilize cell–cell relationships more effectively for clustering, resulting in more precise identification of different cell types and states. The integration of the ZINB model can improve the clustering algorithm’s stability and accuracy. By utilizing the more accurate feature representations provided by the ZINB model and the more accurate graph structure modeling provided by the personalized PageRank algorithm, the clustering algorithm’s sensitivity to noise and disturbances in the data can be reduced, leading to improved clustering stability and accuracy. Lastly, scZAG utilizes a self-optimizing deep embedding clustering approach, feeding the latent features extracted by APPNPGCN into an adaptive clustering module and employing the KL divergence to fulfill the clustering task.

To validate the clustering efficacy of scZAG, we compared it against other state-of-the-art scRNA-seq clustering methods on ten real datasets. Based on the clustering performance, and corroborated by the results of the Mann–Whitney U test, scZAG significantly outperformed the other methods. We also conducted a thorough analysis of hyperparameters to identify the optimal settings for the scZAG approach. Ablation studies confirmed that each component within scZAG positively contributes to the overall performance of the model. Finally, our visualization analysis demonstrated that, compared to other methods, scZAG’s latent embedding representations more effectively differentiate and separate the various cell populations.

When the number of cell types in the dataset increases, both our method and other methods may experience a decrease in accuracy. This is because as the diversity of cell types increases, the complexity of the dataset also increases, making it more challenging to differentiate between different cell types. Additionally, there may be overlapping or similar gene expression patterns among different cell types in the dataset, further complicating accurate classification. In the future, we will continue to improve the balance of scZAG and apply it to the integration of single-cell multi-omics data. Furthermore, we aim to enhance the interpretability of the model by integrating topic modeling techniques.

## 4. Materials and Methods

**Figure 5:**
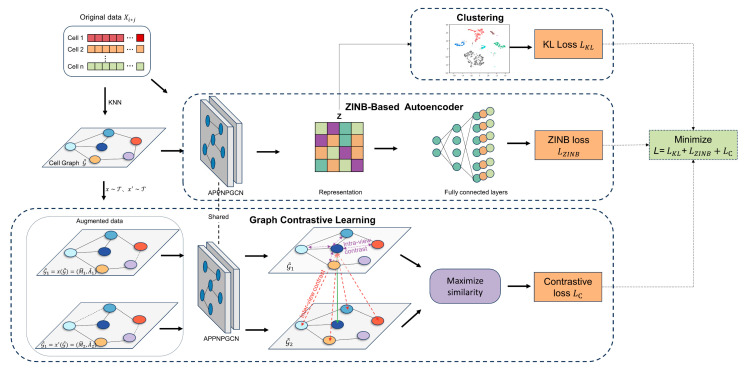
The structural architecture of scZAG. The gene expression matrix is taken as the raw data for scZAG, and a cell graph is constructed via KNN. Subsequently, we generate two augmented cell graphs through random enhancements of the graph structure and attributes. The low-dimensional latent representations of the two enhanced cell graphs are then learned through the APPNPGCN encoder. Next, the model is trained using a contrastive learning objective, aiming to ensure that a node’s representation is similar across two different views yet distinctive from all other nodes’ representations. Note that we define negative samples as all other nodes within the two views. Hence, the negative samples originate from two sources: intra-view (purple) and inter-view nodes (red). scZAG feeds the data $X_{i*j}$ and the cell graph $\tilde{G}$ into a ZINB-based autoencoder to learn the low-dimensional latent representation Z and obtains the ZINB loss through the ZINB decoder. Finally, scZAG clusters the low-dimensional latent representation Z using the Kullback–Leibler (KL) divergence and optimizes clustering by minimizing the KL loss.

The architecture of scZAG is illustrated in Figure 5. scZAG can be divided into three modules: the ZINB-based autoencoder module, the graph contrastive learning module, and the clustering module. First, the ZINB-based autoencoder module employs a ZINB graph convolutional autoencoder to capture the zero-inflation features of scRNA-seq data, further extracting global probability information to reduce noise impact. The graph contrastive learning module introduces a joint adaptive data augmentation strategy targeting topological structures and node attributes, including edge deletion and feature masking. We utilize centrality metrics to identify important edges and feature dimensions. Using the APPNPGCN as the encoder for scZAG, feature extraction is performed on the enhanced cell graphs, considering higher-order neighbor information of nodes to better capture both local and global graph structures. Based on the dimensionality-reduced data, graph contrastive learning effectively captures the topological structures and relationships between nodes in the graph, thus learning more representative node features. The clustering module optimizes the cell clustering process using KL divergence. KL divergence is a method for measuring the difference between two probability distributions, ensuring that during clustering, similar units or nodes are assigned to the same cluster while dissimilar ones are separated. By minimizing KL divergence, we can ensure that the clustering results closely resemble the underlying distribution of the data.

### 4.1. Data Sources

To validate the clustering performance of the scZAG model on scRNA-seq data, we compared it with several state-of-the-art scRNA-seq clustering methods across ten real datasets, which are detailed in Table 2 and originate from recently published papers on scRNA-seq clustering. These datasets come from various sequencing platforms, species, and organs. To assess the clustering performance, we employed two common evaluation metrics: the adjusted Rand index (ARI) and normalized mutual information (NMI), both of which are used to measure the consistency between the generated clusters and the true groups. For both of these evaluation metrics, higher values indicate better clustering performance.

### 4.2. Data Pre-Processing

The scRNA-seq gene expression matrix X is used as the input for our model, where $X_{ij}$ represents the expression count of the *j*th gene (1 ≤ *j* ≤ O) in the *i*th cell (1 ≤ i ≤ N). To ensure the quality and reliability of the data, we employ the following pre-processing methods to pre-process the raw scRNA-seq gene expression matrix. First, quality control and data filtering constitute the initial step of our pre-processing. Taking reference from scGNN [31], we filter out genes that are expressed in more than 1% of cells but are non-zero and genes that are not expressed. Next, as the count matrix data are discrete and subject to large-scale factor variations, we normalize them, followed by rescaling the discrete data using a natural logarithm transformation. The normalization is defined as follows:

(1) $$N\left(X_{ij}\right)=ln\left(m(X)\frac{X_{ij}}{\sum_{o}X_{io}}\right),$$

where m(X) represents the median of the total expression values of the cells. Lastly, we select the top 500 highly variable genes based on the normalized discrete values calculated by the scanpy package [32]. This approach is intended to highlight key variations within the data, thus improving the accuracy and interpretability of further analysis.

### 4.3. Cell Graph

Similar to previous work [13], we use the KNN (K nearest neighbors) algorithm to construct a cell graph from the pre-processed data, where each node in the graph represents a cell. For each cell, we find its K nearest neighbors and connect them. Thus, each cell is connected to its K closest cells, forming a graph. In our experiments, we set the value of K to 15. We use the Euclidean distance to describe the correlation between nodes in order to identify the k shortest distances. Subsequently, the cell graph we construct is undirected, with all edges weighted equally at 1.

### 4.4. Graph Contrastive Learning Framework

The graph contrastive learning framework we employ follows the common graph contrastive learning paradigm, where the model aims to maximize the consistency of representations across different views. Specifically, we begin by generating two graph views through random graph augmentations applied to the input data. We then utilize a contrastive objective that ensures that the encoded embeddings of each node within the two different views remain consistent with each other and distinguishable from the embeddings of other nodes.

In each iteration of scZAG, we employ two random augmentation functions, x∼T and $x^{\prime }∼T$, where T represents the set of all possible augmentation functions. We then obtain two augmented graphs, ${\tilde{G}}_{1}=x(\tilde{G})$ and ${\tilde{G}}_{2}=x^{\prime }(\tilde{G})$, with node embeddings $U=f\left({\tilde{M}}_{1},{\tilde{A}}_{1}\right)$ and $V=f\left({\tilde{M}}_{2},{\tilde{A}}_{2}\right)$, where ${\tilde{M}}_{*}$ represents the feature matrix of the view and ${\tilde{A}}_{*}$ represents the adjacency matrix of the view. A discriminator (contrastive objective) is then employed to distinguish between embeddings of the same node in these two views and embeddings of other nodes. For any node, its embedding $u_{i}$ in one view is the anchor, and its embedding $v_{i}$ in the other view is the “positive sample.” All other embeddings in the two views are treated as negative samples, representing nodes different from the anchor. To facilitate meaningful feature representations, we utilize the InfoNCE multi-view contrastive loss function, defining the pairwise objective for each positive pair as follows:

(2) $$l\left(u_{i},v_{i}\right)=log\frac{e^{\theta \left(u_{i},v_{i}\right)/\tau }}{\underset{\mathrm{intra}-\mathrm{view}\;\mathrm{negative}\;\mathrm{pairs}}{\underbrace{\sum_{k\neq i}e^{\theta \left(u_{i},u_{k}\right)/\tau }}}+\underset{\mathrm{inter}-\mathrm{view}\;\mathrm{negative}\;\mathrm{pairs}}{\underbrace{\sum_{k\neq i}e^{\theta \left(u_{i},v_{k}\right)/\tau }}}+\underset{\mathrm{positive}\;\mathrm{pair}}{\underbrace{e^{\theta \left(u_{i},v_{i}\right)/\tau }}}},$$

where τ is a temperature parameter. We define the critic function $\theta \left(u_{i},v_{i}\right)=s(g(u),g(v))$, where s(⋅,⋅) represents a predetermined similarity function, and g(⋅) is a non-linear projection function, which aims to enhance the expressive power of the critic function. The projection function g is implemented through a two-layer perceptron model. Through this design, the model is able to learn a powerful critic function that can accurately evaluate the similarity of node embeddings in different views, thereby contributing to the achievement of the contrastive learning objective.

For each pair of positive samples, negative samples are defined from both inter-view and intra-view nodes, corresponding to the first and second terms of Equation (2). As the two views are symmetrical, the loss from the alternate view is similarly denoted as $l\left(v_{i},u_{i}\right)$. The overall objective to maximize, representing the average of all positive sample pairs, is defined as follows:

(3) $$J=\frac{1}{2N}\sum_{i=1}^{N}\left[l\left(u_{i},v_{i}\right)+l\left(v_{i},u_{i}\right)\right],$$

In summary, each training round involves applying two random data augmentation functions, x and $x^{\prime }$, to generate augmented graphs, ${\tilde{G}}_{1}=x(\tilde{G})$ and ${\tilde{G}}_{2}=x^{\prime }(\tilde{G})$. Node features within these augmented graphs are learned using a graph convolutional autoencoder based on personalized PageRank propagation, resulting in node embeddings U and V. The model optimizes the objective function in Equation (3) during training, adjusting its parameters to maximize this function and learn node embeddings that effectively capture relationships between nodes.

### 4.5. Adaptive Graph Augmentation

In our scZAG model, we employ an adaptive augmentation approach [20] that preserves important structures and attributes while perturbing less significant edges and nodes. This means that when we randomly delete edges and mask node features, the probability of deletion varies according to the importance of each edge and node. Edges or features with lower importance are more likely to be removed or masked; conversely, those with higher importance have a lower probability of being disrupted. Overall, we emphasize the preservation of important structures and attributes rather than random destruction of the view. This method better guides the model to retain fundamental topological structures and semantic graph patterns.

#### 4.5.1. Topology-Level Augmentation

For the topology-level augmentation, we randomly drop edges from the graph with a bias towards the importance of the edges. Formally, we sample a modified subset $\tilde{A}$ from the original adjacency matrix A with the following probability:

(4) $$P\{\left(u,v\right)\in \tilde{A}\}=1-p_{uv}^{e},$$

where $\tilde{A}$ represents the set of edges in the generated view. The importance of edge $\left(u,v\right)$ denoted by $p_{uv}^{e}$ allows the augmentation function to more likely disrupt edges of lesser importance, ensuring that the generated view maintains critical connectivity structures. Node centrality is employed to assess the prominence of nodes, and we define edge centrality $w_{uv}^{e}$ based on the centrality of the connecting nodes $\left(u,v\right)$. Specifically, $w_{uv}^{e}=\left(\varphi_{c}\left(u\right)\;+\;\varphi_{c}\left(v\right)\right)/2$, where $\varphi_{c}\left(\cdot \right):V⟶R^{+}$ is a node centrality measure.

To assess the likelihood of edge removal based on centrality, we introduce $s_{uv}^{e}=\log\;w_{uv}^{e}$, accounting for varying centrality magnitudes. Subsequently, we normalize centrality values to transform them into probabilities, defined as follows:

(5) $$p_{uv}^{e}=min\left(\frac{s_{max}^{e}-s_{uv}^{e}}{s_{max}^{e}-\mu_{s}^{e}}\cdot p_{e},p_{\tau }\right),$$

where $p_{e}$ is a hyperparameter that governs the overall likelihood of edge deletion, $\mu_{s}^{e}$ and $s_{max}^{e}$ represent the mean and maximum values of $s_{uv}^{e}$, respectively. The term $p_{\tau }<1$ is a cutoff probability that truncates the probability to prevent an excessively high chance of deletion, which would lead to an overly disrupted graph structure.

We define PageRank centrality as the node centrality function. PageRank centrality is determined by the PageRank weights derived from the PageRank algorithm, which disseminates influence across directed edges, and nodes that accumulate the greatest influence are considered important. Formally, the centrality values are computed as follows:

(6) $$\sigma =\alpha AD^{-1}\sigma +1,$$

where $\sigma \in R^{N}$ is the vector of PageRank centrality scores for each node. αis a damping factor that mitigates the absorption of ranks by sink nodes in the graph. Following the recommendation of Lawrence et al. [33], we set the damping factor α to 0.85. Since our cell graph is undirected, we transform it into a directed graph before applying the PageRank algorithm, where each undirected edge is replaced by two directed edges.

#### 4.5.2. Node-Attribute-Level Augmentation

At the node attribute level, we introduce noise to node attributes by randomly masking portions of dimensions in node features with zeros. Formally, we first adopt a random vector $\tilde{m}\in \{0,1{\}}^{F}$, where each dimension is independently drawn from a Bernoulli distribution, i.e., $\tilde{m_{i}}∼\mathrm{Bern}\left(1-p_{i}^{f}\right),\forall i$. The resulting node feature matrix $\tilde{X}$ is:

(7) $$\tilde{X}={\left[x_{1}\circ \bar{m};x_{2}\circ \bar{m};\cdots ;x_{N}\circ \bar{m}\right]}^{⊤},$$

where $\left[\cdot ;\cdot \right]$ denotes the concatenation operation, and ∘ is the element-wise multiplication. Similar to topological enhancement, the probability $p_{i}^{f}$ reflects the importance of that node feature in the ith dimension. We assume that feature dimensions that appear in nodes with greater influence are important and define the weight of feature dimensions as follows. For sparse one-hot node features, we compute the dimension weight of i as follows:

(8) $$w_{i}^{f}=\sum_{u\in V}x_{ui}\cdot \varphi_{c}\left(u\right),$$

where $x_{ui}\in \left\{0,1\right\}$ indicates the presence of feature dimension i in node u, and $\varphi_{c}\left(u\right)$ measures the importance of node u. For dense node features of a node u, we take the absolute value $\left|x_{ui}\right|$ to assess the feature weight in dimension i:

(9) $$w_{i}^{f}=\sum_{u\in V}\left|x_{ui}\right|\cdot \varphi_{c}\left(u\right)$$

Similar to topological enhancement, we then normalize these weights to obtain the importance probabilities for the feature in a given dimension:

(10) $$p_{i}^{f}=min\left(\frac{s_{max}^{f}-s_{i}^{f}}{s_{max}^{f}-\mu_{s}^{f}}\cdot p_{f},p_{\tau }\right),$$

where $s_{i}^{f}=\log\;w_{i}^{f}$. Finally, combining topological enhancement and node attribute enhancement, we generate two augmented views, ${\tilde{G}}_{1}$ and ${\tilde{G}}_{2}$.

### 4.6. Graph Convolution Based on Personalized PageRank Propagation

To better capture the global structural information in graphs while maintaining computational efficiency, we utilize an autoencoder based on approximate personalized propagation of neural predictions using graph convolution (APPNPGCN) [34]. The core idea is to propagate neural predictions through an approximation of personalized PageRank, which is guided by the graph’s edge structure. This helps generate node embeddings that reflect a node’s global position and its neighborhood information within the graph. Using this approach enhances the model’s ability to learn from scRNA-seq data. In the case of standard GCNs, when multiple layers are involved, the mean aggregation approach can lead to over-smoothing issues. Thus, standard GCNs lose the capability to capture local structures. Resorting to larger neighborhoods would inevitably increase the neural network’s depth and the number of learnable parameters.

To overcome the loss of local structure capture, we draw on the connection between the limiting distribution and PageRank. Using a personalized PageRank variant with a root node x, the equation $\pi_{ppr}\left(i_{x}\right)=\left(1-\alpha \right)\dot{\hat{A}}\pi_{ppr}\left(i_{x}\right)+\alpha i_{x}$ computes the distribution of personalized PageRank values starting from the root node x. α is a damping factor determining the likelihood of returning to the root node x for random walk restarts, and $\dot{\hat{A}}$ is the normalized adjacency matrix describing node connectivity. Solving this equation iteratively yields personalized PageRank values, $\pi_{ppr}\left(i_{x}\right)$, for each node i based on the root node *x*. By solving this equation, we can obtain the following:

(11) $$\pi_{ppr}\left(i_{x}\right)=\alpha {\left(I_{n}-\left(1-\alpha \right)\hat{A}\right)}^{-1}i_{x}$$

The indicator vector $i_{x}$ allows us to preserve the local neighborhood of nodes even within the limiting distribution.

We begin with initial predictions based on each node’s unique features and enhance them using personalized PageRank, defining the core concept of neural predictive personalized propagation (APPNP). APPNP employs a power iteration method for efficient topic-sensitive PageRank approximation with linear computational complexity. Unlike traditional PageRank, each power iteration step corresponds to random walks with restarts, improving prediction accuracy. The computation for each power iteration step in the topic-sensitive PageRank variant follows the formulae below:

(12) $$Z^{\left(0\right)}=H=f_{\theta }\left(X\right),$$

(13) $$Z^{\left(k+1\right)}=\left(1-\alpha \right)\hat{A}Z^{\left(k\right)}+\alpha H,$$

(14) $$Z^{\left(K\right)}=\mathrm{softmax}\left(\left(1-\alpha \right)\hat{A}Z^{\left(K-1\right)}+\alpha H\right),$$

where X denotes the feature matrix, $f_{\theta }$ is a neural network with parameter theta that generates predictive results H. The prediction matrix H serves as both the starting vector and the propagation set, with K being the number of iterations, where k∈[0,K−2]. During the process of generating predictions and continuously propagating these predictions, the model undergoes end-to-end training. This means that during back propagation, gradients flow through the propagation scheme (implicitly involving an infinite number of neighborhood aggregation layers). Incorporating these propagation effects can significantly enhance the model’s accuracy.

### 4.7. ZINB-Based Graph Convolutional Autoencoder

To address the issues of excessive zeros and over-dispersion in scRNA-seq data, we incorporate a zero-inflated negative binomial distribution into a graph convolutional autoencoder based on personalized PageRank propagation to learn low-dimensional embeddings of gene expression. The reconstruction of scRNA-seq data using the ZINB-based graph convolutional autoencoder is defined as follows:

(15) $$NB\left(x|\mu ,\theta \right)=\frac{\Gamma \left(x_{i}+\theta \right)}{x_{i}!\Gamma \left(\theta \right)}{\left(\frac{\theta }{\theta +\mu }\right)}^{\theta }{\left(\frac{\mu }{\theta +\mu }\right)}^{x},$$

(16) $$\mathrm{ZINB}\left(X|\pi ,\mu ,\theta \right)=\pi \delta_{0}\left(X\right)+\left(1-\pi \right)\mathrm{NB}\left(X\right),$$

where θ and μ denote the dispersion and mean parameters, respectively, while π represents the zero-inflation probability, which is the likelihood of an observation being zero. To estimate the three critical parameters of the ZINB distribution, μ, θ, and π, we employ three distinct fully connected layers within our computational framework.

(17) $$\pi =sigmoid\left(W_{\pi }f_{D}\left(Z\right)\right),$$

(18) $$\mu =exp\left(W_{\mu }f_{D}\left(Z\right)\right),$$

(19) $$\theta =exp\left(W_{\theta }f_{D}\left(Z\right)\right),$$

where $f_{D}$ is a fully connected neural network comprising three hidden layers with 128, 256, and 512 nodes, respectively. $W_{\pi }$, $W_{\mu }$, and $W_{\theta }$ represent three weight matrices corresponding to the parameters of our model. π, μ, and θ denote the zero-inflation probability, the mean of the negative binomial distribution, and the dispersion parameter, respectively. We utilize the negative log-likelihood of the ZINB distribution as the reconstruction loss for the original data X:

(20) $$L_{\mathrm{ZINB}}=-\log\left(\mathrm{ZINB}\left(X|\pi ,\mu ,\theta \right)\right)$$

### 4.8. Self-Optimizing Deep Graph-Embedded Clustering

Self-optimizing deep embedding clustering integrates graph embedding with deep clustering, aiming to optimize clustering performance by learning non-linear embeddings within the graph structure. Traditional clustering algorithms are unsupervised and label-free, lacking optimization feedback during training. Therefore, we employ self-optimizing deep embedding clustering, which receives optimization feedback throughout the training process, yielding more efficient and accurate results when clustering graph-structured data. We utilize the KL divergence to measure the discrepancy between two probability distributions, *P* and *Q*. Here, *P* represents the true distribution, while *Q* represents the model distribution or an approximation. We define the clustering loss as follows:

(21) $$L_{c}=KL\left(P|Q\right)=\sum_{i}\sum_{u}p_{iu}\log\frac{p_{iu}}{q_{iu}},$$

where $q_{iu}$ is the soft label for the embedded node $z_{i}$. $q_{iu}$ measures the similarity between $z_{i}$ and the cluster center u using a Student’s *t*-distribution and is defined as follows:

(22) $$q_{iu}=\frac{{\left(1+{\left|\left|z_{i}-\mu_{u}\right|\right|}^{2}\right)}^{-1}}{\sum_{r}{\left(1+{\left|\left|z_{i}-\mu_{r}\right|\right|}^{2}\right)}^{-1}}$$

Initial clusters {u} are generated via spectral clustering after pre-training our model. $p_{iu}$ is the auxiliary target distribution, refined to the following:

(23) $$p_{iu}=\frac{q_{iu}^{2}/\sum_{i}q_{iu}}{\sum_{r}\left(q_{ir}^{2}/\sum_{i}q_{ir}\right)}$$

This approach allows the model to iteratively improve the cluster assignments through feedback mechanisms inherently built into the training process.

## Figures and Tables

**Table 1:** Measurement of ablation studies using NMI and ARI metrics. Boldface indicates the best results in the ablation experiments.

Methods	NMI	ARI
GCN + GCA + ZINB	0.836	0.844
APPNPGCN + ZINB	0.847	0.854
APPNPGCN + GCA	0.795	0.809
scZAG	**0.869**	**0.881**

**Table 2:** Summary of the scRNA-seq datasets.

Dataset	Cell	Gene	Class	Platform	Reference
Adam	3660	23,797	8	Drop-seq	[25]
Young	5685	33,658	11	10x	[26]
QS_Limb_Muscle	1090	23,341	6	Smart-seq2	[27]
QS_Heart	4365	23,341	8	Smart-seq2	[27]
QS_Lung	1676	23,341	11	Smart-seq2	[27]
Qx_Limb_Muscle	3909	23,341	6	10x	[27]
Muraro	2122	19,046	9	CEL-seq2	[28]
Wang_Lung	9519	14,561	2	10x	[29]
Guo	6490	27,477	10	10x	[30]
QS_Trachea	1350	23,341	4	Smart-seq2	[27]

## Data Availability

The source code of scZAG has been uploaded to https://github.com/RJiXiang/scZAG, accessed on 27 May 2024. All the datasets used in our experiments can be downloaded from https://cblast.gao-lab.org/download, accessed on 27 May 2024.
